# Supplementary material for: The causal relationship between the human gut microbiota and pyogenic arthritis: a Mendelian randomization study
Source: Front Cell Infect Microbiol. 2024 Nov 26;14:1452480. doi: 10.3389/fcimb.2024.1452480 (PMC11629706; doi:10.3389/fcimb.2024.1452480)
Supplement: Supplementary file 1 [file DataSheet1.zip › mendelian test/ebi-a-GCST90027551.csv_forest.pdf]

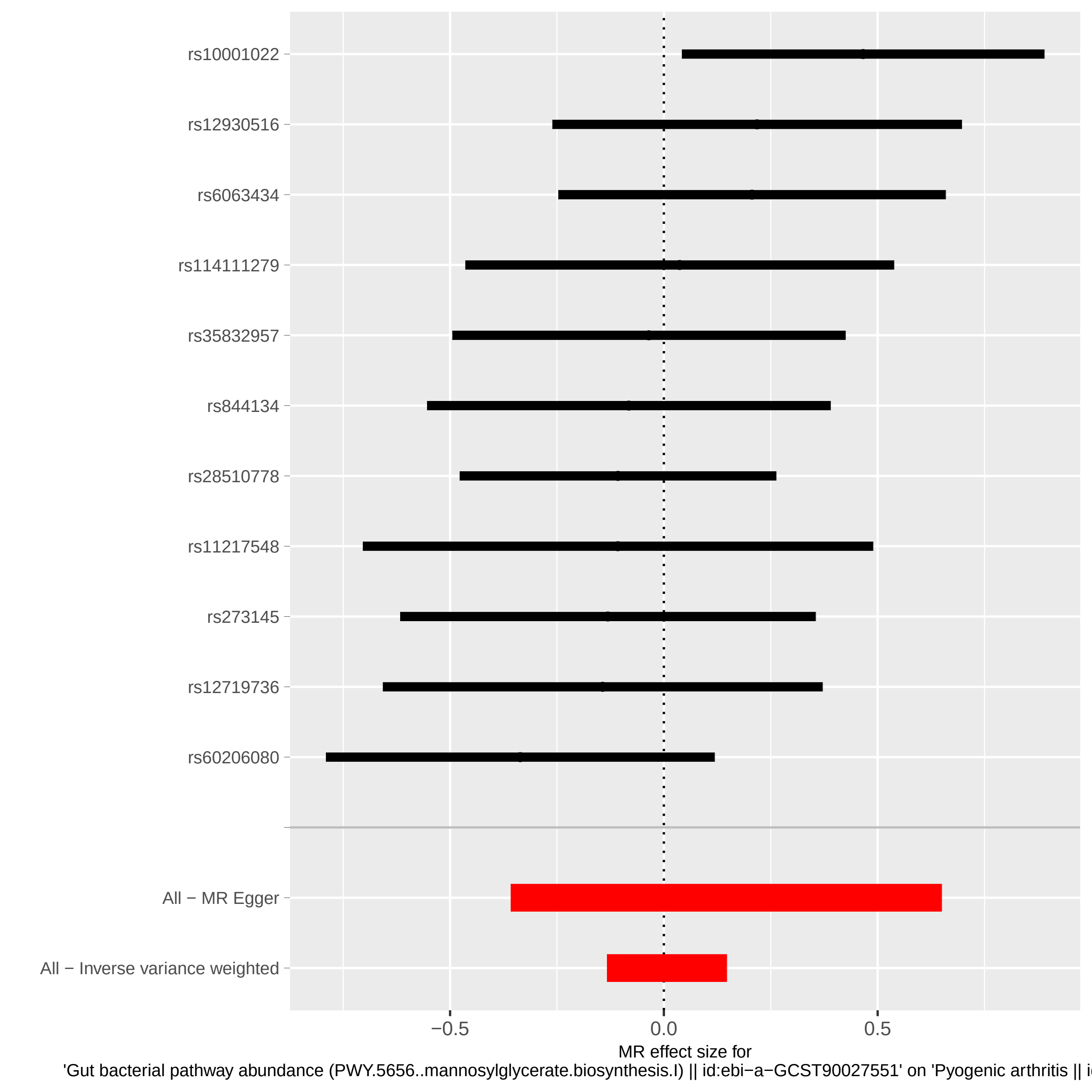

rs10001022
rs12930516
rs6063434
rs114111279
rs35832957
rs844134
rs28510778
rs11217548
rs273145
rs12719736
rs60206080
All − MR Egger
All − Inverse variance weighted
−0.5
0.0
0.5
MR effect size for
'Gut bacterial pathway abundance (PWY.5656..mannosylglycerate.biosynthesis.I) || id:ebi−a−GCST90027551' on 'Pyogenic arthritis || i
